# Burden of paraquat poisoning in the department of Antioquia, Colombia

**DOI:** 10.1186/s40360-019-0291-0

**Published:** 2019-02-15

**Authors:** Jefferson Antonio Buendía, Gabriel Jaime Restrepo Chavarriaga, Andrés F. Zuluaga

**Affiliations:** Grupo de Investigación en farmacología y toxicología, Centro de Información y Estudio de Medicamentos y Tóxicos (CIEMTO), Facultad de Medicina, Universidad de Antioquia, Medellín, Colombia

**Keywords:** Paraquat, Global burden of disease, Colombia, Poisoning, Incidence, Mortality

## Abstract

**Background:**

Paraquat (PQ) poisoning is a public health problem in many regions of Colombia. This study aimed to estimate the burden of PQ poisoning in the Department of Antioquia, Colombia.

**Methods:**

Disability-adjusted life year (DALYs) were calculated as the sum of years of life lost (YLL) and years of life lived with disability (YLD) due to paraquat poisoning in Antioquia; a bootstrapped method with 1000 iterations was used to estimate each statistical parameter using the package DALY calculator in R. For this instance, the annual incidence of paraquat poisoning was obtained from the reported surveillance data according to regional Government.

**Results:**

From 2010 to 2016, 3299 DALYs were estimated in the department of Antioquia for PQ intoxication, with a rate of 53.4 DALYs per 100,000 inhabitants. The majority of the DALYs (2852 DALYs) were generated for men ranging from 15 to 44 years old.

**Conclusion:**

The rate of DALYs reported here is higher than that reported by all chemical poisonings. Better strategies to regulate and restrict the market of this dangerous products are required in Colombia.

## Background

Paraquat (PQ) poisoning is a major health problem worldwide, mainly due to self-poisoning related with suicides or by occupational exposure [1–4]. Globally, 250,000 to 370,000 people die from pesticide poisoning each year, and more than 90% of the individuals with acute poisoning attempted to commit suicide by intentionally ingesting PQ [5]. Most deaths occur in Southeast Asia, Central and South America [1]. The marketing of PQ has been banned in 32 countries; but it low-cost and unrestricted availability promotes its extensive use mainly in rural areas in developing countries [6, 7]. According to the National Institute of Health in Colombia, pesticides resulted in 1231 deaths in the period between 2008 and 2015, with a worrying positive trend accentuated in northeast of this country [8–10]. Paradoxically, the global burden of disease in Colombia never has been determined, in consequence, the mortality or the disability induced by acute PQ intoxications is underestimated and minimized compared with its economic impact on benefit to agricultural production. Then, the lack of pharmacoeconomic indicators preclude the promotion of a better regulatory legislation that prioritize first the risk on the human health over any apparent economic or social benefit [11–13]. In 1990’s, the Global Burden of Disease study quantified the health effects of morbidity and mortality attributable to more than 100 diseases around the world. Since that time, a new more-complex metric was used different than traditional estimates of incidence, prevalence and mortality. These new indicators allow to determine the real burden of the disease by measuring both years of life lost as well as premature death and disability [14]. While the burden of pesticides poisoning has been studied widely, commonly these reports do not differentiate the type of chemical involved in the poisoning [15]. Without any doubt, the disability and lethality of the PQ poisoning is the higher although lesser frequent compared with any of the other pesticides intoxications, then pharmacoeconomic studies in this area are limited and potentially biased [16]. A valid and consistent description of burden of disease is a great input to generate better health-policies and planning processes. Here, the aim was to estimate the disease burden of PQ poisoning in the Department of Antioquia (Colombia) between 2010 to 2016.

## Methods

To estimate the PQ burden of disease we use hospital admissions and mortality rates from comprehensive data for Department of Antioquia. This department is the second department with the highest population of the country (around 6,613,118 inhabitants) [17].

All records of patients intoxicated by PQ during the 2010–2016 in the department of Antioquia to the national epidemiological surveillance system (SIVIGILA) were analyzed. In SIVIGILA, all patients intoxicated by chemical substances are reported online or in paper form to the SIVIGILA by each hospitals; being this report obligatory and made by personnel trained. The information recorded is age, sex, ingestion at hospital admission, amount ingested, symptoms, ethnicity, and type of exposure and mortality.

No personally identifiable information was recorded all information obtained from health surveillance systems were kept confidential. Consent was not required because this was a study that used secondary sources of information already published. This study was approved by the Institutional Review Board of University of Antioquia (2015–4690).

The mortality data were in turn contrasted with that reported by the National Department of Statistics - DANE for the department in the period studied. We calculated the DALY for acute poisoning using methods described by Murray and Lopez in the GBD study [18]; which summed the YLL and YLD components. The basic formula is expressed as follows: DALY = YLL + YLD. We estimated YLL by multiplying the number of PQ poisoning deaths by the number of years of expected remaining life at the respective age of death according to reference life tables of the global burden of disease study [11]. For all estimates were used the population structure of the department of Antioquia in 2013 (half of the study period) [17]. Next, the YLD was obtained by multiplying the number of acute poisoning incidents by both the average duration of poisonings (The values of duration of the disease and rate of remission for acute poisoning and paraquat-induced pulmonary fibrosis were obtained from the literature [19–21]) as an acute cause of death/disability and a disability weight that reflected the severity of poisonings on a scale from 0 (perfect health) to 1 (death) derived from the 2015 GBD study (Disability weights used for acute poisoning was 0.163 (95% CI 0.109–0.227) and pulmonary fibrosis was 0.019 (95% CI 0.011–0.033) [5, 15]. We did not consider co-morbidities due to lack of data and lack of the possibility of follow-up due to the retrospective manner of the study. We did not consider the severity of poisoning in terms of disability for the same reason, and no particular disability weight was given according to the severity of the case. The internal consistency of each parameter was evaluated using the DISMOD II program (38); following the recommendations and methodology described in the manual for national studies of WHO disease burden [22].

### Statistical analysis

Given the inherent variability in using epidemiological data as a source of information for the estimation of the DALYs, a probabilistic sensitivity analysis of each of the parameters incorporated into the DALY estimation model was made. Each epidemiological parameters were accompanied by a confidence or credibility interval, or represented as a probability distribution, rather than being represented by a single point estimate. Monte Carlo simulations is a technique was used to incorporate this uncertainty in the DALYs [23]. In our study all incidences, mortality, duration of disease were set to beta-pert distribution while onset of the disease was set using uniform distribution using the uncertainty intervals estimated with DisMod-II. Then the final estimations, with their confidence intervals, of the Years of Adjusted Life by Disability (DALYS), years of life lost due to premature death (YLL) and years of life lived with disability (YLD) were made by performing 1000 iterations in a Monte Carlo simulation of each parameter using bootstrapped technique of DALY calculator package in R program [24]. All estimates are performed using a discount rate of 3% and weighting by age (C = 0.1658, β = 0.04). Subgroup analyzes were performed according to age group and sex. The probabilistic sensitivity analysis was performed using the entire range of initial values of each of the parameters used to calculate the DALYs using the standardized regression coefficient method. The simulated overall DALY estimates were regressed against the simulated values for the stochastic input parameters. To facilitate comparison, the independent terms are standardized such that they were normally distributed with mean zero and standard deviation one. The resulting regression coefficients were therefore referred to as standardized regression coefficients. The regression coefficients correspond to the number of standard deviations change in overall DALY given one standard deviation change in the corresponding input parameter.

## Results

In total, 154 patients with diagnosis of PQ poisoning were reported from department of Antioquia, between: 2010 to 2016.to the national epidemiological surveillance system (SIVIGILA). The majorities were mestizos and white, farmworkers, most of them between 20 and 29 years, coming of Uraba, with intentional exposure per oral route. The incidence rate was 2.37 per 100,000 inhabitants (CI 95%: 2.14–2.95 per 100,000 patients). Highest incidence rates were seen males (2.78 per 100,000 inhabitants), farmworkers, between 30 to 39 years old, respect to females (1.96 per 100,000 inhabitants) or males of other age groups. 14 deaths were registered to the national epidemiological surveillance system. Highest mortality was seen in males, between 30 to 39 years old, with intentional ingestion of PQ, see Table 1.

**Table 1:** Incidence of PQ poisoning per 100,000 cases

Sex/Age	2010	2011	2012	2013	2014	2015	2016
Men							
< 20	0000	0179	0179	0179	0179	0000	0447
20–29	0616	0616	1847	1436	1026	1026	0821
30–39	0259	0000	1036	0259	1036	1036	0518
40–49	0217	0434	0434	0000	0867	0000	0217
> 50	0000	0167	0167	0000	0167	0334	0501
Females							
< 20	0279	0279	0186	0652	0559	0466	0652
20–29	0396	0396	1187	0396	0396	0989	1187
30–39	0472	0000	0472	0472	0236	0236	0472
40–49	0000	0000	0000	0000	0213	0213	0000
> 50	0000	0000	0000	0000	0134	0000	0000

3299 DALYs (p2.5-p97: 3299–3423 ALYs) were estimated, with DALYs rate of 0.534 DALYs per 1000 inhabitants. 99% of the DALYs were caused by YLL and only 1% by YLD. Most of the DALYs (2852 DALYS) were generated between 15 and 44 years of age. About 57% of the DALYs were in men, see Table 2. Acute PQ poisoning generated 99% of the DALYs (3349), whereas PQ fibrosis only generated 13 DALYs.

**Table 2:** Distribution by sex and age of DALYs, YLL, YLD

	DALYS		YLD		YLL	
Age	Men	Female	Men	Female	Men	Female
0–4	0	43	0	0	0	43
5–14	82	0	0	0	82	0
15–44	1582	1280	9	1	1573	1279
45–59	221	135	0	0	221	134
60+	17	0	0	0	17	0

Years of Adjusted Life by Disability (DALYS), years of life lost due to premature death (YLL) and years of life lived with disability (YLD)

**Fig. 1:**
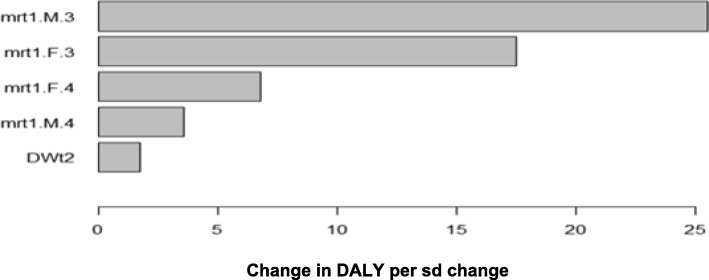
Sensibility analysis. mrt1.M3: men’s mortality 15–44 years, mrt1.F3: female’s mortality 15–44 years, mrt1.F4: female’s mortality 45–59 years, mrt1.M4: men’s mortality 45–59 years, DWt2: disability weights of PQ acute intoxication

The results were robust in the sensitivity analysis, the percentage of change in the total estimate of DALYS did not exceed 25% with the variables analyzed; being the probability of death due to acute intoxication in men and women between 15 and 44 years the variable associated with the highest percentage of change in the DALYs (between 5 and 25%, of the final estimate). There were no significant variations between the discount rate between 0 to 5%, Table 3 and Fig. 1.

**Table 3:** Results of sensitivity analysis: Mapped standardized regression coefficients

Input	Sex	Age	Estimate	Std.Error	t value	Pr(>|t|)
Mortality	men	15–44 years	25.601	0.005	4.794.598	0000
Mortality	female	15–44 years	17.473	0.005	3.272.977	0000
Mortality	female	45–59 years	6.818	0.005	1.277.255	0000
Mortality	men	45–59 years	3.524	0.005	660.125	0000
Disability weights of PQ pulmonary fibrosis			1.741	0.005	325.981	0000
Mortality	men	5–14 years	1.241	0.005	232.429	0000
Mortality	female	0–4 years	0.872	0.005	163.254	0000
Mortality	men	60+ years	0.352	0.005	65.986	0000
Disability weights of PQ acute intoxication			0.185	0.005	34.735	0000
Incidence rate	men	15–44 years	0.157	0.005	29.346	0002
Incidence rate	female	15–44 years	0.017	0.005	3.097	0001
Incidence rate	female	60+ years	0.013	0.005	2.342	0001
Incidence rate	men	0–4 years	−0.012	0.005	−2.333	0001
Incidence rate	female	5–14 years	0.012	0.005	2.172	0029
Incidence rate	female	45–59 years	− 0.009	0.005	−1.747	0086
Incidence rate	men	45–59 years	−0.009	0.005	−1.712	0086
Incidence rate	men	5–14 years	0.008	0.005	1.484	0.138
Incidence rate	men	60+ years	−0.008	0.005	−1.446	0.148
Incidence rate	men	15–44 years	0.008	0.005	1.424	0.154
Incidence rate	men	5–14 years	0.008	0.005	1.418	0.156
Incidence rate	men	45–59 years	0.007	0.005	1.295	0.195
Disease duration	female	15–44 years	0.007	0.005	1.219	0.223
Disease duration	men	15–44 years	0.006	0.005	1.104	0.269
Disease duration	female	5–14 years	0.006	0.005	1.066	0.287
Incidence rate	female	0–4 years	−0.005	0.005	−0.976	0.329
Disease duration	female	45–59 years	0.004	0.005	0.778	0.436
Incidence rate	female	15–44 years	0.004	0.005	0.754	0.451
Age at disease onset	men	0–4 years	−0.004	0.005	−0.750	0.453
Age at disease onset	female	60+ years	−0.003	0.005	−0.603	0.546
Age at disease onset	men	60+ years	0.003	0.005	0.549	0.583
Disease duration	men	5–14 years	0.002	0.005	0.467	0.641
Disease duration	men	0–4 years	−0.002	0.005	−0.411	0.681
Incidence	female	45–59 years	0.002	0.005	0.389	0.697
Incidence	female	0–4 years	0.002	0.005	0.361	0.718
Age at disease onset	men	5–14 years	−0.001	0.005	−0.112	0.91
Incidence rate	men	60+ years	−0.001	0.005	−0.104	0.917

## Discussion

The burden of disease in the northeast of Colombia by PQ poisoning was around 53.4 years of life adjusted for disability per 100,000 inhabitants. In our knowledge, this is the first estimate in terms of burden of the disease specifically for this product. Previous studies have estimated only DALYs for poisoning in general and not by each chemical substance. For example, global burden study estimated that all poisonings account around 3,149,000 DALYs, and in Colombia: 6021 DALYs (12.41 DALYs per 100,000 inhabitants) [25]. In relative terms, all poisoning generate only 0.059% of the total number of DALYs in our country; behind the violence, traffic accident, and self-injurious injuries (8.85, 3.53 and 1.6% of the country’s total DALYS, respectively). These estimations contrast with our results, where the rate of DALYS per PQ is four times higher than all poisonings (53.4 per 100,000 inhabitants’ vs 12.41 DALYs per 100,000 inhabitants). These differences may be due to different sources of information used in the first place; while global burden of disease, used data from national health survey to estimate the incidence and mortality data; our source was directly the records of epidemiological surveillance for incidence. The great advantage of using the records of epidemiological surveillance is that they have a greater degree of completeness and less probability of information biases since they are mandatory records in our country and completed directly by the doctors who treats the poisoning; and not a survey which may be subject to information bias due to its retrospective nature. In this regard, similar behavior is also observed when comparing the rates of DALYs between the global burden of disease study and the study of burden of the disease in Colombia in 2010. National study used SIVIGILA data -as in ours study- and rates are obtained in some cases also higher than reported by the global burden of disease study. For example, the global burden study of the disease estimates a total of 21,191 DALYs per 100,000 for Colombia; while the Colombian study of burden of disease 2015 [15] estimates a higher total value of around 26,900 DALYs per 100,000; this behavior is accentuated especially in injuries generated from external causes where the most used sources corresponded to SIVIGILA. This higher estimate based on specific epidemiological surveillance data was also observed in a study performed in Iran, where the rate of DALYs was higher than that reported by the global burden of disease study, even observing an incremental trend with the time opposite to that posed by the global study [26].

In our study, most DALYs (86%) were generated by men between 15 and 44 years of age; This data correlates with other studies, for example in Iran [26], global and national estimates of disease burden [15, 25], and epidemiological studies of suicidal behavior with chemical substances; where higher rates were observed in young adult male farmers [1]. Another finding is the relative importance of PQ poisoning compared to other more well-known diseases. For example, PQ poisoning alone in this region is above – in terms of DALYs- of common pathologies such as meningitis or sexually transmitted diseases except HIV (56 vs 28 DALYs × 100,000 inhabitants) [14, 25, 27, 28] . The differences in this case in addition to the source of information already discussed, are caused in this because unlike non-HIV sexually transmitted diseases where the vast majority of the DALYS (71%) are generated by YLD. In PQ poisoning, 99% are due to premature deaths in young adults, which have more weight in estimating the DALYs - YLL are not weighted by disability weights- which according to the pathology reduce their impact in the total calculation of DALYs. This reflects the usefulness of the DALYs to estimate the potential impact of a disease on the population, where pathologies such as externally caused injuries continue to be preventable health problems in underdeveloped countries where regulatory and health education strategies should be prioritized.

Our study has the following limitations: regarding the possible information bias due to the use of retrospective information and despite the fact that the case report of poisoning by chemical substances is mandatory for all institutions in the country; some degree of underestimation in cases of poisoning by PQ is possible. However, in the department of Antioquia there has been a tendency since 2008 to increase the reporting of intoxication cases to SIVIGILA, which may reduce the risk of this information bias [29]. To minimize the possible information bias due to the use of probability values extracted from the literature, which come from populations other than the Colombian population; sensitivity analysis was performed for each of these parameters taking a range of possible values and their distribution. As it was evidenced the final result of DALYs was not sensitive to the change in the values of said probabilities guaranteeing the robustness of the model. Second, there are no specific “disability weights” for PQ intoxication or for PQ-induced pulmonary fibrosis. In our study, we used those reported for intoxications in general and for moderate COPD based on the fact that the disability generated by pulmonary fibrosis, at least in terms of spirometry and in survival is not very different from that presented by patients with moderate COPD, as evidenced in previous studies both local [30] and international [20]. In the sensitivity analysis, the percentage of change in the total estimate of DALYS did not exceed 25% with the variables analyzed, achieving the greatest change with the mortality rates of men and women between 15 and 44 years. Mortality in this study was extrapolated from the literature given because there are still serious limitations in the death records in our country regarding the death cause. It is not uncommon for PQ intoxication or any chemical substance to be placed as a cause of death, with which mortality rates may undervalue a true estimate. Also we do not consider the exclusion of patients with other concomitant poisonings since it was not a variable that could be validated in the registries.

Our result show that PQ poisoning is a serious problem, with a great social impact; which requires national policies that regulate the controlled use of these substances or their prohibition In this regard in countries as Korea, the PQ ban is associated with a reduced mortality rate of herbicide poisoning. After the PQ ban, the number of associated poisonings decreased, while that of the other less toxic herbicides poisonings increased. It may also have resulted from decreased intentional ingestion [7].

In US, PQ is not banned, but is use solely by certified applicators. In Europe, PQ is prohibited in Sweden, Finland, Norway, Germany and Netherlands [2]. The reality in Colombia and in most developing countries is different, where PQ is marketed without restriction. The responsibility for suicidal use of PQ rests also on the manufacturer. Unrestricted access to a liquid, of which a very small amount may be fatal, makes a suicidal decision easy. Regulatory agencies have not fully recognized either the inherent toxicity of PQ for human beings or the particular risks derived from exposures in developing countries. For medical systems our results may lead to the generation of risk management policies prioritizing strategies for the prevention of suicide with PQ in young people in order to mitigate the impact of such poisoning in our countries. It is clear that the reduction in the DALYs depends in our country of the reduction of the mortality rate in young people and adults. This rate depends not only on effective prevention and promotion policies, but also improvements in the quality of medical care. Our results show this problematic unknown until now, and are supported with local data which constitutes an added value with respect to other studies.

## Conclusions

The rate of DALYs reported here is higher than that reported by all chemical poisonings. Better strategies to regulate and restrict the market of this dangerous products are required in Colombia. Further studies are needed to evaluate potentially modifiable factors associated with the suicide attempt with PQ, especially in the young and farmer population, where the highest burden of disease is presented by this condition.

## Abbreviations

DALYs: Disability-adjusted life year
GBD: Global burden of disease study
PQ: Paraquat
SIVIGILA: National epidemiological surveillance system
YLD: Years of life lived with disability
YLL: Years of life lost
